# Progress in Obstetrics

**Published:** 1895-11-09

**Authors:** 


### PROGRESS IN OBSTETRICS.

**Abdominal Section in Puerperal Peritonitis.**—Noble[1] discusses the indications for this operation, and comes to the following conclusion. This operation does not hold out the least prospect of cure in ordinary cases of acute general lymphatic peritonitis, because, although the peritoneal cavity may be freely irrigated and drained, it is impossible to get at or remove the infective agent. It may be called for in cases of localised purulent peritonitis, but it is nearly always limited to cases of peritonitis due to rupture, bruising or twisting of an ovarian cyst, and to those cases where pus, which existed previously in the tubes or ovaries, has been set free and so excited general peritonitis. The operation of vaginal hysterectomy in cases of septic endometritis following abortion or childbirth is not likely to find many advocates, because if the infection is limited to the uterus it is quite unnecessary, and in cases where the infection is not limited to the uterus it is absolutely useless. Baldy[2] relates two cases in which he performed this operation after a miscarriage; in each case the infection had spread into the broad ligaments, and both the patients died.

**The Use of the Curette in Cases of Puerperal Infection.**—It is still an open question if it is desirable to use the curette in complications following full-term delivery. Demelin[3] states that the chief indication is infection, but he also recommends curettage in cases where a portion of the placenta is retained and has begun to undergo decomposition. He further asserts that in febrile conditions associated with retention of a thickened and hypertrophied decidua the curette should be used if the fever does not yield in three or four days to uterine irrigation. Perrin[4] describes fourteen cases treated in this manner by Auvard with the object of demonstrating that curettage of the uterus is the most certain means of obtaining rapidly a fall of temperature. He confesses, however, that the fall of temperature is not immediate, and that usually on the first night after the operation the temperature is higher than before. He then says that on the next day, though sometimes not for two or three days, the fever disappears. The value of this paper is much diminished by the fact that the majority of the cases narrated are cases of fever arising after abortion, and not after full-time delivery. Dumont[5] points out the dangers and complications that may arise from curettage of a recently-delivered uterus; he finds that it is impossible to remove the whole of the infected tissue in cases of septic endometritis following childbirth, because of the size of the cavity and the softness of the uterine wall, and thus the curette may be the means of opening the door to general infection. Severe hæmorrhage occasionally follows the operation, and in some cases the uterine wall has been perforated.

Lusk[6] strongly condemns the use of the curette in cases of endometritis following child-birth. He asserts that these cases will get perfectly well if let alone, and that by the uncalled-for use of the curette and intra-uterine douche severe and fatal complications may arise, and this is probably the experience of anyone who is constantly called on to see and deal with these cases.

**Symphysiotomy.**—It is probable that before long the value of this operation will be more fully recognised in this country, as up to the present its difficulties and dangers have been much exaggerated. The chief obstacles to its popularity in England up to the present have been (1) the supposed difficulty in performing the operation in the patient's own house; (2) the fear that the division of the symphysis may afterwards interfere with the patient's power of locomotion. Greater familiarity with the operation will do much to dispel the former objection, and experience goes to show that the fear of bad after-results is unfounded. The work of Pinard[7] and his colleagues is that which demands the greatest attention. In 1894 this operation was performed twenty-two times out of 2,147 cases. Of these twenty-two there were three maternal deaths, and two of these deaths were attributed to septicæmia contracted before admission. In none of the cases was any special difficulty encountered, nor any serious hæmorrhage or injury to the soft parts, and thirteen of the patients were primiparæ. In none of these nineteen cases were any unpleasant after-effects noticed, and in one case the operation was performed a second time. If the head is delayed by pelvic contraction, provided the contraction is not too great, this operation is always performed at this institution, forceps, version, and craniotomy having been abandoned. In 1892 eighty-five cases were reported with the loss of ten women and twenty-four children. In 1893, 148 cases were reported, with the loss of eighteen women and twenty-nine children.

**Rupture of the Uterus.**—Dr. Edgar[8] writes about a case that was brought under his notice. For five hours she was under the care of a midwife, who stated she gave no ergot, and only made two vaginal examinations. She was then handed over to the care of a physician, who made several unsuccessful attempts to deliver by version, and next morning sent the patient into the hospital. No difficulty was then found in turning and delivering, but the placenta failing to appear an examination was made, and revealed a utero-vaginal rupture of the genital tract. The umbilical cord was then followed up, and the placenta removed from among the intestines in the peritoneal cavity.

The woman died two hours and forty minutes after delivery, and it was found that death was due to internal concealed hæmorrhage, for several pints of fresh blood were found in the abdominal cavity, and the hæmorrhage had evidently come from the left uterine artery, which had been completely torn across. This case illustrates the hopelessness of expectant treatment of uterine rupture by gauze drainage, where the rupture has involved the uterine artery, and where internal hæmorrhage has been going on into the peritoneal cavity.

As a rule, in these cases laparotomy only adds to the shock previously sustained, and the patient is almost invariably lost. Statistics from the Vienna Maternity Hospital show this very plainly. They have saved about 50 per cent. of the patients treated on the expectant plan. The haphazard introduction of gauze is quite likely to cause further tearing of the uterus, which is generally in these cases in a friable condition, and unless the packing is carefully and systematically done one can hardly expect it to be an efficient means of controlling the uterine hæmorrhage. Dr. Robert Murray speaks strongly against immediate laparotomy in these cases, notwithstanding the fact that most of the text-books lay it down as the usual and proper method of treatment. The mortality by any treatment appears to be about 75 per cent.

[1] Pract., Aug., 1895. [2] Amer. Gynæcol. and Obst. Journ., April, 1895. [3] Arch. de To:ol et de Gynécol., March, 1895. [4] Ditto. March, 1895. [5] Ditto. March, 1895. [6] Amer. Gynæcol and Obst. Journ , April, 1895. [7] Brit. Med. Journ., Aug. 10, 1895. Annal. de Gynæcol., Jan., 1895. [8] N.Y. Med. Journ., May 5, 1895.

